# Ventral Tegmental Area GABA Neurons Are Resistant to GABA(A) Receptor-Mediated Inhibition During Ethanol Withdrawal

**DOI:** 10.3389/fnins.2018.00131

**Published:** 2018-03-05

**Authors:** Ashley C. Nelson, Stephanie B. Williams, Stephanie S. Pistorius, Hyun J. Park, Taylor J. Woodward, Andrew J. Payne, J. Daniel Obray, Samuel I. Shin, Jennifer K. Mabey, Scott C. Steffensen

**Affiliations:** Department of Psychology and Center for Neuroscience, Brigham Young University, Provo, UT, United States

**Keywords:** VTA, GABA, neurons, muscimol, GABA(A) receptor, alcohol, withdrawal

## Abstract

The neural mechanisms underlying alcohol dependence are not well-understood. GABAergic neurons in the ventral tegmental area (VTA) are a relevant target for ethanol. They are inhibited by ethanol at physiologically-relevant levels *in vivo* and display marked hyperexcitability during withdrawal. In the present study, we examined the effects of the GABA(A) receptor agonist muscimol on VTA neurons *ex vivo* following withdrawal from acute and chronic ethanol exposure. We used standard cell-attached mode electrophysiology in the slice preparation to evaluate the effects of muscimol on VTA GABA neuron firing rate following exposure to acute and chronic ethanol in male CD-1 GAD-67 GFP mice. In the acute condition, the effect of muscimol on VTA neurons was evaluated 24 h and 7 days after a single *in vivo* dose of saline or ethanol. In the chronic condition, the effect of muscimol on VTA neurons was evaluated 24 h and 7 days after either 2 weeks of twice-daily IP ethanol or saline or following exposure to chronic intermittent ethanol (CIE) vapor or air for 3 weeks. VTA GABA neuron firing rate was more sensitive to muscimol than DA neuron firing rate. VTA GABA neurons, but not DA neurons, were resistant to the inhibitory effects of muscimol recorded 24 h after a single ethanol injection or chronic ethanol exposure. Administration of the NMDA receptor antagonist MK-801 before ethanol injection restored the sensitivity of VTA GABA neurons to muscimol inhibition. Seven days after ethanol exposure, VTA GABA neuron firing rate was again susceptible to muscimol's inhibitory effects in the acute condition, but the resistance persisted in the chronic condition. These findings suggest that VTA GABA neurons exclusively undergo a shift in GABA(A) receptor function following acute and chronic exposure. There appears to be transient GABA(A) receptor-mediated plasticity after a single exposure to ethanol that is mediated by NMDA glutamate receptors. In addition, the resistance to muscimol inhibition in VTA GABA neurons persists in the dependent condition, which may contribute to the the hyperexcitability of VTA GABA neurons and inhibition of VTA DA neurons during withdrawal as well as the motivation to seek alcohol.

## Introduction

Alcoholism is a chronic relapsing disorder that has an enormous impact on society. A major goal of research on alcoholism is to characterize the critical neural substrates that are most sensitive to alcohol, adapt in association with chronic consumption and drive subsequent alcohol-seeking behavior. The mesocorticolimbic dopamine (DA) system originating in the ventral tegmental area (VTA) of the midbrain and projecting to the nucleus accumbens (NAc) is known to be involved in reward. The emerging view is that the dysregulated homeostasis that accompanies the development of drug addiction may result from experience-dependent neuroadaptations that hijack normal synaptic transmission in this system (Hyman and Malenka, 2001; Hyman et al., 2006; Kauer and Malenka, 2007; Nugent and Kauer, 2008).

The VTA is highly involved in adaptive reward and motivation processing and is composed of DA (65%), γ-aminobutyric acid (GABA; 30%), and glutamate (GLU; 5%) neurons (Dobi et al., 2010). Both *in vivo* (Gessa et al., 1985) and *in vitro* (Brodie et al., 1990; Brodie and Appel, 1998) electrophysiological studies indicate that acute ethanol increases VTA DA neuron firing rate (EC_50_ of 120 mM in the slice) and DA release in limbic structures (Imperato and Di Chiara, 1986), and that withdrawal from chronic ethanol reduces DA firing rate and release in the NAc (Diana et al., 1993). Although the release of DA in the NAc positively reinforces drug use, it has been suggested that the changes in DA are strongly regulated by VTA GABA neurons (Nugent and Kauer, 2008; Steffensen et al., 2011; Ting and van der Kooy, 2012; Bocklisch et al., 2013). GABAergic projections to the VTA come from several regions including the NAc, rostro-medial tegmentum, and the ventral pallidum, but another major inhibitory regulation of VTA DA and GABA neurons is by GABAergic interneurons within the VTA (Johnson and North, 1992; Steffensen et al., 1998). In support of this, results from studies in Cre mice [GAD-Cre (Tan et al., 2012) or VGAT-Cre (van Zessen et al., 2012)] expressing channel rhodopsin-2 show that selective activation of VTA GABA neurons by light stimulation inhibits DA neuron activity, inducing conditioned place aversion (Tan et al., 2012) and disrupting reward consumption (van Zessen et al., 2012). In contrast, silencing the activity of VTA GABA neurons by expressing the proton pump halorhodopsin in VTA GABA neurons disinhibits DA neurons (Bocklisch et al., 2013). We have shown in multiple reports that acute ethanol inhibits the firing rate of VTA GABA neurons in rats with an IC_50_ of 1.0 g/kg (Gallegos et al., 1999; Stobbs et al., 2004; Ludlow et al., 2009; Steffensen et al., 2009), which is one order of magnitude more sensitive than ethanol effects on DA neurons (Gysling and Wang, 1983; Mereu et al., 1987; Brodie and Appel, 1998). VTA GABA neurons are even more sensitive to ethanol in C57BL/6 and CD-1 mice, as they are inhibited with an IC_50_ of 0.25 g/kg (Steffensen et al., 2011). Of most relevance to this study, VTA GABA neurons recorded *in vivo* become hyperexcitable (firing rates averaging >100 Hz) during withdrawal from ethanol and tolerance accrues to ethanol inhibition of VTA GABA neuron firing rate (Gallegos et al., 1999). Thus, VTA GABA neurons undergo pronounced adaptation during withdrawal from chronic ethanol.

We have shown in multiple reports that GABA(A) receptors [GABA(A)Rs] switch their function during opiate dependence (Laviolette et al., 2004; Vargas-Perez et al., 2009, 2014; Ting-A-Kee et al., 2013). The functional switch results from increased levels of brain-derived neurotrophic factor (BDNF), which activates the high-affinity tyrosine kinase B (TrkB) receptor (Vargas-Perez et al., 2009), which is expressed in VTA GABA neurons (Numan et al., 1998). Ethanol withdrawal not only produces adaptations in VTA GABA neurons (Gallegos et al., 1999), but also GABA(A)R subunit composition in the VTA and the hippocampus (Charlton et al., 1997; Cagetti et al., 2003), and it is reasonable to assume that these changes are important for precipitating this switch in the neurobiological substrates mediating ethanol reinforcement. Considerable evidence suggests that activation of GABA(A)R complexes can produce depolarization in lieu of its more traditional hyperpolarizing response (Kaila et al., 1993; Staley et al., 1995; Rivera et al., 1999; Hübner et al., 2001; Coull et al., 2003). The switching of GABA(A)R functionality occurs during development and under pathological conditions like epilepsy. There is a major gap in our understanding of the neural substrates that adapt with alcohol dependence, and whether or not they are causal or reflective. Thus, the aim of this study was to evaluate GABA(A)R function in VTA GABA neurons during withdrawal from acute and chronic ethanol. We hypothesized that, similar to what we have reported with opiate dependence, VTA GABA neuron GABA(A)R sensitivity to the GABA(A)R agonist muscimol would adapt to chronic ethanol, reflecting a functional shift of the receptors and VTA GABA neurons themselves during withdrawal.

## Materials and methods

### Animal subjects

Male glutamate-decarboxylase-67 (GAD-67) green fluorescent protein (GFP) knock-in CD-1 (white albino) mice (Tamamaki et al., 2003) were bred and cared for in accordance with the National Institutes of Health (NIH) Guide for the Care and Use of Laboratory Animals. For each methodology employed, animals were treated in strict accordance with the Brigham Young University Animal Research Committee (IACUC) guidelines, which reviewed and approved the procedures detailed herein. Once weaned at post-natal day 21, all mice were housed in maximum groups of four and given ad libitum access to solid food and water and placed on a reverse light/dark cycle with lights ON from 8 p.m. to 8 a.m. Any mice used in injection experiments were briefly (2–5 min) anesthetized with isoflurane to reduce the stress of the injection and allow for administration of large volumes, and injected intraperitoneally (IP) with a sterile needle. Animals returned to their home cages 30 min following an injection.

### Chronic intermittent ethanol exposure to establish alcohol dependence

Animals were made dependent on ethanol in one of two methods of chronic intermittent ethanol (CIE) exposure, either by multiple injections or in vapor chambers. In the multiple injections method, mice were injected IP with ethanol (3.0 g/kg) or saline twice-daily for 14 days, which was sufficient to establish dependence, as we have reported previously (Gallegos et al., 1999). However, no attempt was made in injection studies to determine dependence (i.e., increased drinking). In the vapor chamber method, ethanol vapor was used to establish ethanol dependence. We modified the vapor chamber system developed in the lab of Graeme Mason at Yale (Wang et al., 2012). The six automated chamber system consisted of an air-pressurized, feedback-controlled ethanol flask with flow valves to each of three sealed chambers to regulate the flow of air (11 L/min) and concentration of alcohol to three of six chambers placed in a ventilation hood. A breathalyzer (Drager Alcotest 6510) was used in a feedback loop to regulate the concentration of ethanol. In order to avoid overdosing the first week of CIE vapor exposure, mice were exposed to 4, 6, and 8 h of vapor before exposing to 16 h vapor/day. Control animals were housed in three sealed chambers in the same ventilation hood, but only received air. Blood alcohol levels (BALs) were measured using the an enzymatic kit (Sigma-Aldrich, St. Louis, MO). Even at the 5 L/min feedback flowmeter level the air-exposed mice did not show any detectable alcohol above the 1 mg% detection limit of the breathalyzer or the BAL determination method.

### Drink-in-the-dark behavioral experiments

To observe a behavioral correlate of alcohol dependence and validate our vapor chamber method, mice were trained and evaluated in a drink-in-the-dark (DID) two-bottle choice alcohol drinking test. Mice were exposed to CIE vapor or air for 3 weeks, as described above. After a withdrawal period of 24 h, animals were removed from home cages 3 h into the dark cycle, and placed individually in cages with the bedding and food removed. They were given two sipper tubes, with one containing tap water and the other containing tap water and ethanol (20% v/v). Mice were allowed to drink from the tubes for 2 h in the dark, and were then returned to their home cages. They repeated this test over 5 consecutive days with no CIE vapor or air exposure during that time. Animals then underwent 3 days of withdrawal, and had a 1-day challenge DID session on day 9 with identical conditions.

### Preparation of brain slices

All brain slice preparations were performed in P18-120 day old GAD-67 GFP CD1 mice in order to visualize GAD-67+ neurons in the VTA by GFP imaging. P18-28 day old mice were used in the naïve experiements (see results below) only. Ethanol was only administered in animals older than day P28. All mice used in ethanol exposure groups were age-matched and were P28-120 days old with a median age at day P55. There was no effect of age on any results in this study. Brains were rapidly extracted under isoflurane anesthesia. Upon extraction, the brain was glued onto a cutting stage. The brain was then sectioned in ice-cold cutting solution (in mM: 194 Sucrose, 30 NaCl, 4.5 KCl, 1 MgCl_2_, 26 NaH_2_CO_3_, 1.2 NaH_2_PO_4_, 10 Glucose) bubbled with 95% O_2_/5% CO_2_. Targeting the VTA, 210 μm thick horizontal slices were sectioned on a vibratome and then placed in an incubation chamber containing artificial cerebral spinal fluid (ACSF; in mM: 124 NaCl, 3 KCl, 1.25 NaH_2_PO_4_, 26 NaHCO_3_, 12 glucose, 1.5 MgSO_4_, 2 CaCl_2_) bubbled with 95% O_2_/5% CO_2_. After a recovery period of at least 30 min, brain slices were placed in a recording tissue chamber with ACSF continuously flowing at 35°C.

### Cell-attached recording of spike activity in brain slices

Cell-attached mode studies used electrodes pulled from borosilicate glass capillaries (2.5–6 MΩ) and then filled with a NaCl solution containing (in mM) 124 NaCl, 2 KCl, 1.25 NaH_2_PO_4_, 26 NaHCO_3_, 1.2 MgSO_4_, 2 CaCl_2_ adjusted to pH 7.4 with KOH. GABA neurons were identified by fluorescence in GAD-67 GFP mice. Fluorescent cells were imaged on a Nikon Eclipse FNI microscope with a 40x/0.80 n.a. objective lens. The filter cube for GFP detection was a Nikon C-FL ENDOW GFP 96343 cube (Bandpass: 450–490 nm; Barrier: 500–550 nm; dichroic: 495 nm). Excitation was performed with a Sutter Lambda TLED transmitted light source at 506 nm. Cells were then imaged using differential interference contrast imaging in order to facilitate cell attached recordings. Neurons that did no fluoresce and were characterized by relatively slow, regular firing activity were assumed to be DA neurons. Positive pressure was applied to the electrode when approaching the neuron. By applying suction to the electrode, a seal (10 MΩ–1 GΩ) was created between the cell membrane and the recording pipette. Spontaneous spike activity was then recorded in cell-attached mode with an Axon Instruments Multiclamp 700B amplifier, sampled at 10 kHz using an Axon 1440A digitizer, and collected and analyzed using pClamp10 software. Neurons were not clamped throughout these experiments although recorded in voltage clamp mode (voltage-clamp was set to 0 mV). Firing rate recordings in this study were performed in cell-attached mode in order to avoid dialysing the contents of the cells and disrupting the cytoplasmic milieu (e.g., the Cl^−^ ion gradient), which we have shown previously is perturbed with opiate dependence (Ting-A-Kee et al., 2013; Vargas-Perez et al., 2014). A stable baseline recording of firing activity was obtained for 5–10 min before adding drugs. Neurons that did not achieve a stable baseline firing rate during this time were rejected from the study. Muscimol (0.01, 0.1, 1.0, 10.0 μM) in ACSF was perfused in successive doses for 3–5 min at each dose until the neuron stopped firing. ACSF was then applied for 10 min. On a given experimental day, 2–3 horizontal slices containing the VTA were sectioned from a mouse. VTA neurons were recorded and analyzed with at least four mice/group.

### Drug preparation and administration

Muscimol (Sigma-Aldrich) was solubilized in ACSF and superfused on brain slices at 0.01, 0.1, 1.0, and 10.0 μM. Drugs used for injections were solubilized in sterile 0.9% saline and injected IP: Ethanol (16% v/v solution; 3.0 or 4.0 g/kg) and MK-801 (0.5 mg/kg; Sigma-Aldrich, St. Louis, MO).

### Statistical analyses

For neuronal firing rate, results are presented as percent of baseline firing rate ± standard error of the mean (SEM). Statistical significance required ≥ 95% level of confidence (*p* ≤ 0.05). Firing rate was analyzed for 2 min (baseline) before muscimol perfusion and for 2 min at the end (peak effect) of the drug application, or the last 2 min before any drug was applied. For comparison between groups, a mixed model ANOVA was used. Values were Greenhouse-Geisser corrected for sphericity. Data was found to be reasonably distributed using the Wilks-Shapiro test of normality. Using the criterion of median plus or minus 3 interquartile range (IQR) outlying data points were identified. After determining that the outliers were not due to data input error they were bounded to the median ± 3 IQR for analysis purposes. A priori hypothesis testing was accomplished with Bonferroni correction *post-hoc* tests. Analysis software included Minianalysis (Synaptosoft, Decatur, GA), Clampfit (Molecular Devices, Sunnyvale, CA), Microsoft Excel, STATA (StataCorp, College Station, TX), and Igor Pro (Wavemetrics, Oswego, OR). Significance levels were indicated on graphs with asterisks ^*^,^**^,^***^, corresponding to significance levels *p* < 0.05, 0.01, and 0.001, respectively. Figures were constructed with Igor Pro software.

## Results

### Sensitivity of VTA neuron firing rate to the GABA(A) receptor agonist muscimol

**Figure 1:**
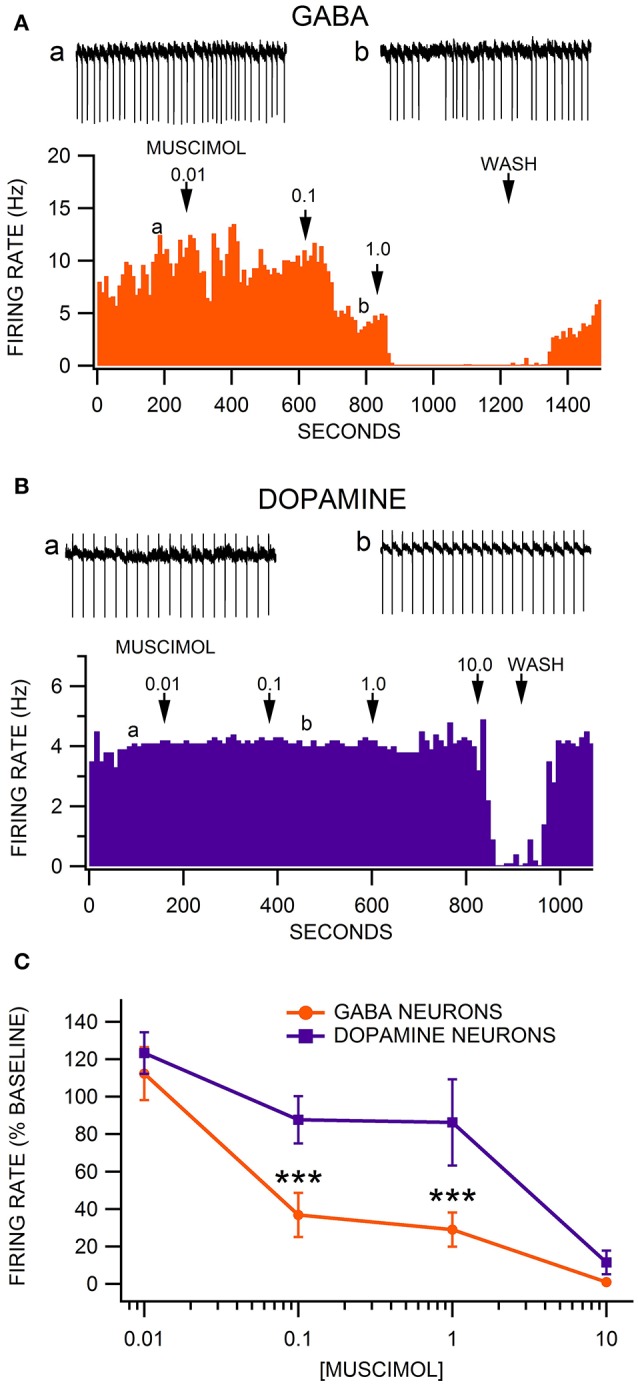
Comparison of the sensitivity of VTA GABA vs. DA neurons to muscimol inhibition of firing rate. **(A)** This representative VTA GABA neuron had a baseline firing rate of ~10 Hz. The GABA(A)R agonist muscimol mildly inhibited the firing rate of this neuron at 0.01 μM, moderately inhibited it at 0.1 μM, and abolished its activity at 1.0 μM. Insets a,b are 5 s traces of GABA neuron spike activity recorded at the times indicated on the graph. **(B)** The ratemeter shows the activity of a representative VTA DA neuron, which had a baseline firing rate of ~4 Hz. Muscimol had little or no effect on this DA neuron until the firing rate was suppressed at 10.0 μM. Insets a,b are 5 s traces of DA neuron spike activity recorded at the times indicated on the graph. **(C)** The firing rate of VTA GABA neurons was significantly more sensitive than VTA DA neurons to muscimol at 0.1 and 1.0 μM. Asterisks ^***^ represent significance levels *p* < 0.001.

We have previously shown that VTA GABA neurons are sensitive to the GABA(A)R agonist muscimol at sub-micromolar concentrations (Ting-A-Kee et al., 2013; Vargas-Perez et al., 2014). We present data on firing rate of putative VTA DA neurons along with VTA GABA neurons in GAD-67 GFP mice. Neurons in the VTA of GAD-67 GFP mice that did not exhibit fluorescence, but were characterized by relatively slow, regular firing activity, were presumed to be DA neurons. The firing rate of VTA GABA neurons in ethanol-naïve animals was significantly faster than putative VTA DA neurons [*F*_(1, 35)_ = 18.91, *p* = 0.0001; GABA neurons = 12.9 ± 2.0 Hz vs. DA neurons = 4.8 ± 0.9 Hz; *n* = 14, 7]. We evaluated the effects of muscimol on VTA neuron firing rate at concentrations ranging from 0.01 to 10.0 μM. Muscimol dose-dependently reduced the firing rate of all neurons tested [*F*_(4, 55)_ = 36.56, *p* < 0.0001]. As reported previously (Ting-A-Kee et al., 2013; Vargas-Perez et al., 2014), superfusion of muscimol inhibited VTA GABA neuron firing rate with an IC_50_ < 0.1 μM (Figure 1A). However, muscimol inhibited VTA DA neuron firing rate with an IC_50_ > 1.0 μM (Figure 1B). In ethanol naïve mice, VTA GABA neurons were significantly more sensitive than VTA DA neurons to muscimol in the 0.1–10 μM range [Figure 1C; *F*_(4, 55)_ = 3.76, *p* = 0.0090]. A priori hypothesis testing with Bonferroni correction revealed that GABA neuron firing rate was more depressed than DA neuron firing rate by 0.1 μM muscimol [*F*_(1, 90)_ = 15.36, *p* = 0.001] and 1.0 μM muscimol [*F*_(1, 90)_ = 22.42, *p* < 0.001; 0.01 μM: *n* = 10, 7; 0.1 μM: *n* = 10, 7; 1.0 μM: *n* = 13, 6; 10.0 μM: *n* = 10, 5].

### Muscimol inhibition of VTA neuron firing rate after a single injection of ethanol

**Figure 2:**
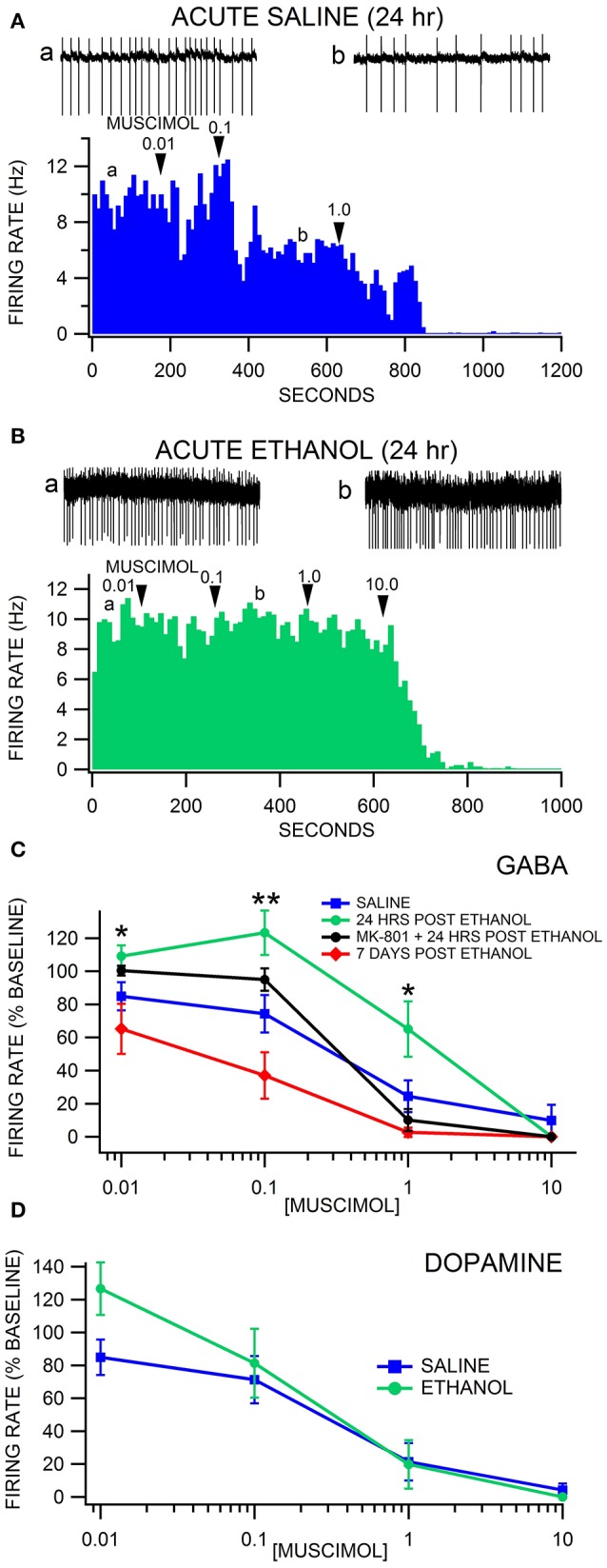
Decreased sensitivity of VTA GABA neurons to muscimol 24 h after a single injection of ethanol. **(A)** The ratemeter shows the firing rate of a VTA GABA neuron (traces in a, b recorded at times indicated on graph) recorded in a brain slice from a mouse injected with saline once, 24 h before recording. This representative neuron had a baseline firing rate of ~9.3 Hz. Muscimol (0.01–10.0 μM) inhibited the firing rate of this VTA GABA neuron. All insets are representative 5 s traces of GABA neuron spike activity recorded before (inset a) and after (inset b) 0.1 μM muscimol. **(B)** The ratemeter shows the firing rate of a GABA neuron, which had a baseline firing rate of ~9.6 Hz. Insets a,b show representative 5 s traces before and after application of muscimol in a mouse that was treated with 4.0 g/kg ethanol once, 24 h before recording. This mouse was resistant to muscimol's inhibitory effects up to 1.0 μM. **(C)** Muscimol significantly inhibited the firing rate of VTA GABA neurons in saline-injected animals. Muscimol's inhibitory effect was significantly reduced 24 h after a single injection of ethanol, but sensitivity was restored by administration of MK-801. Sensitivity to muscimol was restored in mice tested 7 days post ethanol injection. **(D)** Dopamine neurons were equally sensitive to muscimol 24 h after an ethanol injection in ethanol-exposed vs. air-exposed controls. Asterisks ^*^,^**^ represent significance levels *p* < 0.05 and *p* < 0.01 between ethanol and saline groups.

Firing rate was recorded in VTA GABA and DA neurons 24 h after acute saline or ethanol administration (non-dependent condition; Figures 2A,B). GAD-67 GFP mice were administered saline or 4.0 g/kg ethanol IP, which produced sedation for 1–2 h with complete recovery in all mice. In a subset of mice, the NMDA antagonist MK-801 (0.5 mg/kg) was administered IP 15 min before ethanol or saline. There was no significant difference in baseline firing rate between any of the acute treatment groups [*F*_(3, 37)_ = 1.786, *p* = 0.167; Figures 2A–C]. Muscimol depressed VTA GABA firing rate in a dose dependent manner [*F*_(4, 119)_ = 60.76, *p* < 0.0001; Figures 2A–C]. Acute withdrawal from a single exposure of ethanol altered the response of VTA GABA neurons to muscimol [*F*_(15, 119)_ = 3.69, *p* = 0.001; Figures 2B,C]. *Post-hoc* analysis with Bonferroni correction revealed that muscimol suppressed VTA GABA neuron firing rate more in 7 day ethanol withdrawn mice than in 24 h withdrawn mice [*t*_(166)_ = 3.81, *p* = 0.008; Figure 2C] or MK-801 pretreated control mice [*t*_(166)_ = −3.79, *p* = 0.008]. VTA GABA neuron firing rate in 24 h withdrawn ethanol mice was less sensitive to muscimol depression of firing rate than saline mice at 0.1 μM muscimol [*t*_(166)_ = 3.95, *p* = 0.004]. At 1.0 μM muscimol, MK-801 pretreated control mice were less sensitive to muscimol inhibition of GABA neuron firing rate than MK-801 pretreated ethanol withdrawn mice [*t*_(166)_ = 4.01, *p* = 0.004; *n* = 8 each], 7 day withdrawn ethanol-injected mice [*t*_(166)_ = −4.08, *p* = 0.004; *n* = 8 each], or saline-injected mice [*t*_(166)_ = −4.39, *p* < 0.004; *n* = 8 each]. Specifically at 0.1 μM muscimol, VTA GABA neuron firing was affected more by muscimol in 7 day withdrawn mice from a single ethanol injection than in MK-801 (0.5 mg/kg IP administered 15 min prior to ethanol or saline) pretreated 24 h withdrawn mice [*t*_(166)_ = −4.04, *p* = 0.004; *n* = 9 each], MK-801 pretreated control mice [*t*_(166)_ = −4.48, *p* < 0.004], or 24 h withdrawn ethanol mice [*t*_(166)_ = 6.30, *p* < 0.004; Figure 2C].

Firing rate was also recorded in putative VTA DA neurons (GAD-67 GFP-negative cells) 24 h after acute saline or ethanol administration. Muscimol suppressed DA neuron firing rate in a dose dependent manner [*F*_(4, 28)_ = 18.19, *p* = 0.0003; Figure 2D]. A single *in vivo* ethanol exposure did not affect DA neuron firing rate [*F*_(1, 9)_ = 1.41, *p* = 0.2655]. There was no main effect of withdrawal from a single *in vivo* exposure to ethanol on sensitivity to muscimol suppression of DA neuron firing rate [*F*_(3, 28)_ = 1.52, *p* = 0.2491].

### Muscimol inhibition of VTA neuron firing rate after chronic ethanol

**Figure 3:**
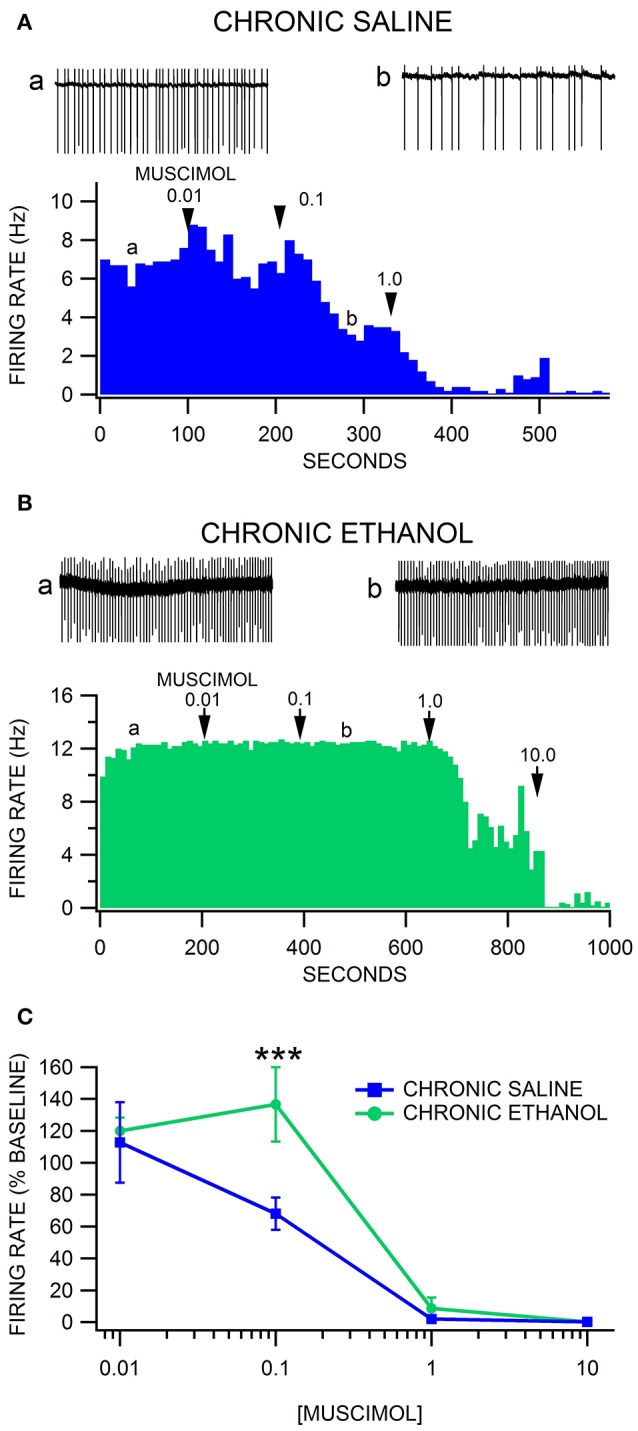
Decreased sensitivity of VTA GABA neurons to muscimol after chronic ethanol injections. **(A)** The ratemeter shows the firing rate of a VTA GABA neuron (traces in a,b recorded at times indicated on graph) recorded in a brain slice from a mouse injected with saline twice-daily for 14 days. This representative neuron had a baseline firing rate of ~7 Hz. Muscimol markedly inhibited the firing rate of this VTA GABA neuron, with suppression of activity at the 1.0 μM concentration. All insets are representative 5 s traces of GABA neuron spike activity recorded before (inset a) and after (inset b) 0.1 μM muscimol. **(B)** The ratemeter shows the firing rate of a GABA neuron in a mouse that was treated with 3.0 g/kg ethanol twice daily for 14 days. The baseline firing rate was ~12 Hz. This mouse was resistant to muscimol's inhibitory effects until it was inhibited by 1.0 μM muscimol. **(C)** Muscimol inhibited the firing rate of VTA GABA neurons in chronic saline-treated animals, which was significantly reduced in chronic ethanol-treated mice. Asterisks ^***^ represent significance levels *p* < 0.001.

We have reported previously that VTA GABA neurons recorded in freely-behaving rats evince hyperexcitability and tolerance to acute ethanol following 2 weeks of twice-daily injections of ethanol (Gallegos et al., 1999). The hyperexcitability of VTA GABA neurons was optimal 16–24 h after withdrawal from the last injection. We used similar methods for establishing alcohol dependence in GAD-67 GFP mice. Similar to what we reported in rats, we observed that most ethanol-injected mice exhibited more agitation, less grooming, rigid tail and piloerection than the saline-injected mice 24 h after 2 weeks of twice-daily injections of 3.0 g/kg ethanol. Unlike what we found previously in rats *in vivo*, there was no difference in baseline VTA GABA neuron firing rate in the slice preparation *ex vivo* in ethanol-treated mice compared to saline-treated mice [*F*_(1, 12)_ = 0.1, *p* = 0.76; Ethanol = 8.8 ± 1.6 Hz vs. Saline = 7.9 ± 0.9 Hz; *n* = 8, 5]. Muscimol depressed VTA GABA firing rate in a dose-dependent manner [*F*_(4, 37)_ = 65.66, *p* < 0.0001; Figure 3]. Chronic ethanol injections significantly affected the response of VTA GABA neurons to muscimol [*F*_(4, 37)_ = 3.82, *p* = 0.0466] recorded 24 h after the last ethanol injection. A priori hypothesis testing with Bonferroni correction revealed that VTA GABA neuron firing rate differed significantly between chronic saline vs. ethanol injected mice at 0.1 μM muscimol in chronic ethanol-treated mice (Figures 3B,C; *n* = 7), evincing relative resistance to muscimol inhibition of VTA GABA neuron firing rate [*F*_(1, 48)_ = 18.97, *p* = 0.0004; Figures 3A,C; *n* = 5].

**Figure 4:**
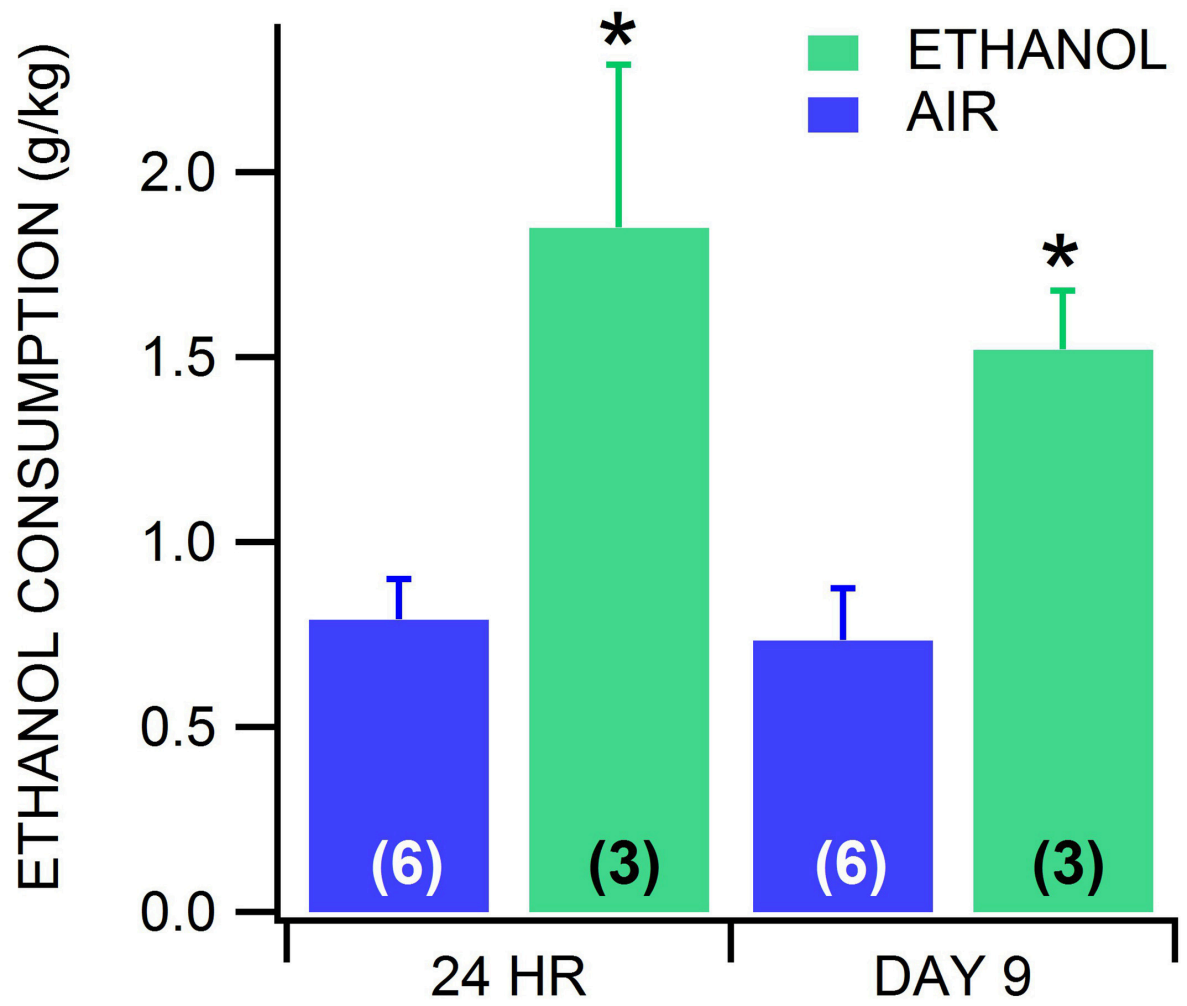
Drink-in-the-dark model of alcohol dependence. In CD-1 GAD-67 GFP mice exposed to three continuous weeks of CIE vapor (*n* = 3) or air (*n* = 6), ethanol consumption in the DID test was significantly increased in mice receiving ethanol vapors vs. air-exposed mice. Dependent mice displayed increased drinking 24 h after withdrawal from CIE vapor, and again on day 9 after last CIE vapor exposure. On day 9, mice were in 72 h withdrawal following the last drinking test performed. Asterisks ^*^ mark statistical significance (*p* < 0.05) between air and CIE vapor groups.

The effects of alcohol dependence on the sensitivity of VTA neurons to muscimol was also evaluated with CIE vapor exposure (200 mg% for 16 h/day) and drink-in-the-dark (DID) ethanol consumption in GAD-67 GFP mice. Mice exposed to ethanol vapors increase their consumption of alcohol when dependent (Lopez and Becker, 2005; Dhaher et al., 2008). To validate the utility of our modified vapor chamber system in producing alcohol dependence with CIE exposure in GAD-67 GFP mice, we calibrated three feedback flowmeter settings (1, 3, and 5 L/min) in a 3 L alcohol pressurized flask with breathalyzer values and blood alcohol levels (BALs; Sigma Enzymatic method). We obtained breathalyzer values of 52.1 ± 4.2, 130.2 ± 4.8, and 225.6 ± 4.1 mg% (*n* = 6) ethanol and BALs of 46.1 ± 3.7, 119.7 ± 2.9, and 234.9 ± 5.5 mg% (*n* = 6) ethanol at feedback flowmeter flask settings 1, 3, and 5 L/min, respectively. Based on these calibrations, a separate cohort of mice was exposed to feedback flowmeter settings corresponding to 200 mg% breathalyzer values for 16 h (1000–0200 h)/day. We evaluated ethanol consumption in the DID procedure following 3 weeks of continuous CIE or air in order to avoid any alcohol exposure in the air-exposed mice. Ethanol vapor-exposed GAD-67 GFP mice consumed significantly more ethanol than air-exposed mice 24 h after withdrawal from 3 weeks of CIE [*F*_(1, 11)_ = 6.9, *p* = 0.02; *n* = 6, 8], as well as 9 days after withdrawal from CIE [*F*_(1, 5)_ = 5.94, *p* = 0.02; *n* = 3 each; Figure 4]. In addition, similar to chronic ethanol injections, we observed that most ethanol-exposed mice exhibited more agitation, less grooming, rigid tail and piloerection than the saline-injected mice 24 h after 3 weeks of ethanol vapor exposure. Thus, 3 weeks of CIE in our vapor chambers at the 200 mg% breathalyzer level was used to evaluate the sensitivity of VTA neurons to muscimol in separate cohorts.

**Figure 5:**
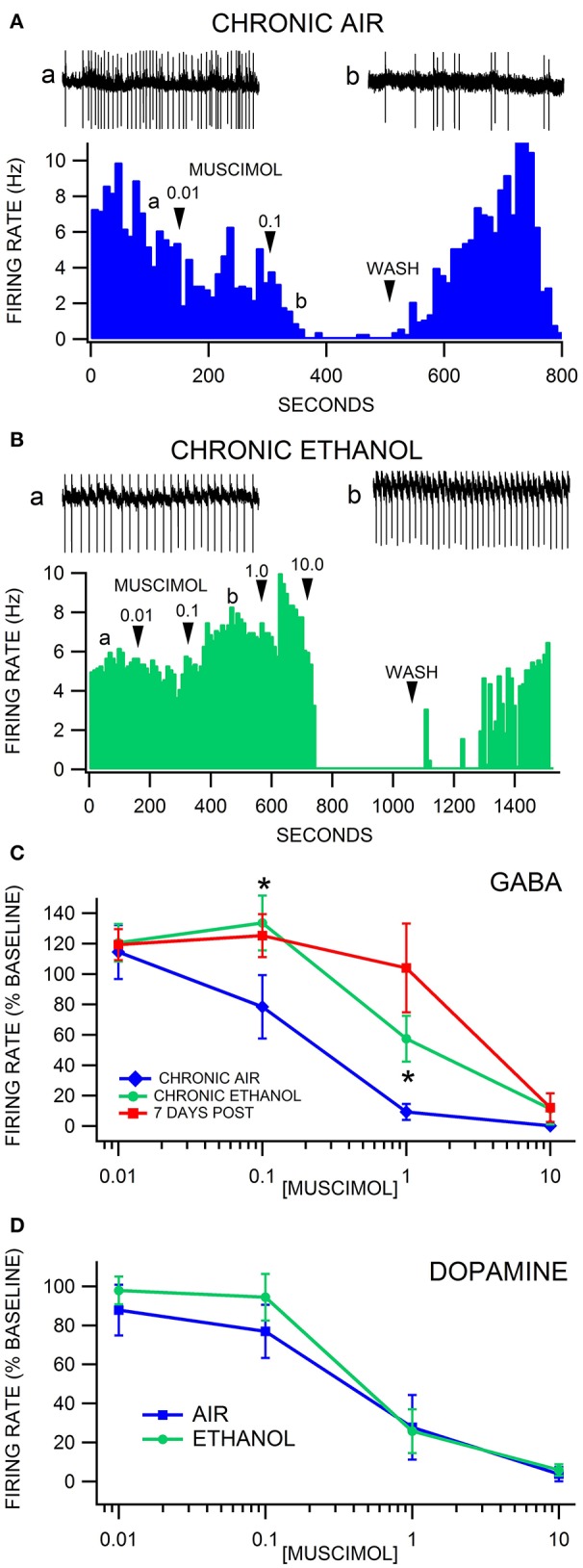
Decreased sensitivity of VTA GABA, but not DA neurons, to muscimol after chronic intermittent ethanol vapor exposure. **(A)** The ratemeter shows the firing rate of a VTA GABA neuron (traces a, b recorded at times indicated on graph) recorded in a brain slice from a mouse exposed to air for 3 weeks. This representative VTA GABA neuron was characterized by irregular activity but with a baseline firing rate of ~7 Hz. The firing rate of this neuron was inhibited by 0.01 μM and suppressed by 0.1 μM muscimol. All insets are representative 5 s traces of GABA neuron spike activity recorded before (inset a) and after (inset b) 0.1 μM muscimol. **(B)** The ratemeter shows the firing rate of a GABA neuron in a mouse that was exposed to CIE vapor for 3 weeks. The baseline firing rate was ~5 Hz. This VTA GABA neuron was more resistant to muscimol's inhibitory effects up to 1.0 μM than its air-exposed control. In fact, the firing rate of this neuron was mildly enhanced by muscimol at the 0.1 and 1.0 μM concentrations. **(C)** Muscimol significantly inhibited the firing rate of VTA GABA neurons in animals exposed to air for 3 weeks, which was significantly reduced in mice exposed to CIE vapors for 3 weeks. In addition, VTA GABA neuron firing rate was resistant to muscimol inhibition after 7 days of withdrawal. **(D)** Dopamine neurons were equally sensitive to muscimol in ethanol vapor-exposed mice vs. air-exposed controls. Asterisks ^*^ represent significance level *p* < 0.05 between ethanol and air groups.

Firing rate was evaluated in VTA neurons 24 h after 3 weeks of CIE to alcohol vapor vs. air controls. Similar to chronic ethanol injections, there was no difference in baseline VTA GABA neuron firing rate in the slice preparation *ex vivo* in CIE vapor-exposed mice compared to air-exposed mice [*F*_(1, 44)_ = 0.02, *p* = 0.89; Ethanol = 9.6 ± 1.1 Hz vs. Air = 9.4 ± 1.0 Hz *n* = 26, 19]. Muscimol depressed VTA GABA neuron firing rate in a dose-dependent manner [*F*_(4, 137)_ = 27.03, *p* < 0.0001]. Treatment condition altered the response of VTA GABA neurons to muscimol [*F*_(8, 137)_ = 4.71, *p* = 0.0014]. A priori hypothesis testing with Bonferroni correction revealed that VTA GABA neuron firing rate was higher in chronic ethanol animals (Figures 5B,C) compared to chronic air animals (Figures 5A,C) when the slices were perfused with 0.1 μM muscimol (*F*_(1, 202)_ = 9.93, *p* = 0.019; *n* = 15, 13] and 1.0 μM muscimol [*F*_(1, 202)_ = 9.92, *p* = 0.019; *n* = 19, 14]. Additionally, VTA GABA neurons in ethanol-dependent mice after 7 days of withdrawal discharged at a higher rate than in chronic air mice during perfusion with 1.0 μM muscimol [*F*_(1, 202)_ = 26.27, *p* < 0.001; *n* = 15, 7].

Firing rate was also evaluated in VTA DA neurons 24 h after CIE vapor exposure vs. air exposure. Dopamine neuron firing rate was unaffected by chronic ethanol exposure [*F*_(1, 21)_ = 0.15, *p* = 0.7003]. Muscimol decreased DA neuron firing rate in a dose dependent manner [*F*_(4, 56)_ = 34.63, *p* < 0.0001]. Chronic ethanol exposure did not affect muscimol inhibition of DA neuron firing rate [*F*_(4, 56)_ = 0.56, *p* = 0.4854; Figure 5D; *n* = 14, 9].

## Discussion

The overall aim of this study was to investigate the electrophysiological components of the mechanism underlying the hyperexcitability of VTA GABA neurons following withdrawal from chronic ethanol. Mainly, we have reported previously that VTA GABA neuron firing rate often exceeds 100 Hz for 4–8 h during withdrawal from chronic ethanol in rats (Gallegos et al., 1999). This is 4-5X the baseline level of VTA GABA neuron firing rate in naïve rats. In addition, tolerance accrues to ethanol inhibition of VTA GABA neurons firing rate following 11 days of chronic ethanol exposure. The focus of this study was to determine the role of GABA(A)Rs in VTA GABA neuron hyperexcitability produced by acute and chronic ethanol exposure in GAD-67 GFP mice, similar to what we have described with opiate dependence (Ting-A-Kee et al., 2013; Vargas-Perez et al., 2014). VTA GABA neurons are resistant to the GABA(A)R agonist muscimol following withdrawal from exposure to acute and chronic ethanol, which was not exhibited in putative VTA DA neurons.

Putative DA neurons in the VTA were not as sensitive to muscimol as VTA GABA neurons. Muscimol binds to the orthosteric site (i.e., where GABA binds) on the α1 subunit of the GABA(A)R. A possible explanation for the decreased sensitivity is that the α1 subunit is selectively expressed in GABA neurons (Fritschy and Mohler, 1995), but not in DA neurons, in the VTA (Okada et al., 2004; Tan et al., 2010), and has been implicated in addiction to some benzodiazepines (Tan et al., 2010). Interestingly, consistent with what has been reported by us and others (Tan et al., 2010), VTA GABA neurons exhibit much more spontaneous and spike-related GABA inhibitory input than DA neurons. This phenomenon occurs despite the fact that GABA neurons have faster firing rates than DA neurons both *in vivo* and *in vitro*, which is operational even with pronounced afferent GABAergic drive. The effects of acute and chronic ethanol on muscimol effects on putative DA neurons were studied along with GABA neurons. There are ambiguities associated with their identification in GAD-67 GFP mice, as 5% of the neurons in the VTA are neither GABA nor DA neurons (Margolis et al., 2012), and some GABA neurons express GAD-65 (Merrill et al., 2015). Thus, while VTA GABA neurons in GAD-67 GFP mice can be unequivocably identified as GAD-67+ neurons, caution must be taken in the identification of DA neurons in this study. However, since DA neurons represent 65% of and GABA neurons 30% of VTA neurons (Margolis et al., 2012), it is highly likely that the GFP-negative neurons were DA neurons.

We show that VTA GABA neurons are less sensitive to the inhibitory effects of muscimol 24 h after a single *in vivo* injection. We were originally surprised by this finding, because we had hypothesized that chronic ethanol exposure would be required to shift the sensitivity of VTA GABA neurons to muscimol, as we have demonstrated with opiate dependence. However, this short-term plasticity was blocked by prior administration of the NMDAR antagonist MK-801 (0.5 mg/kg), suggesting that NMDAR activation is required for acute ethanol's effect on VTA GABA neuron muscimol sensitivity. Because the mechanism is GLU-dependent, at least with acute ethanol exposure, there must be some connection between NMDAR activation and the altered function of GABA(A)Rs on VTA GABA neurons. In support of this, GLU plasticity on VTA DA neurons has been shown to be induced by GABA(A)R agonists(Vashchinkina et al., 2012, 2014). Future studies could address interactions between GLU and GABA plasticity, or a link between acute NMDAR activation and an increase in intracellular chloride concentration, which would shift the reversal potential of the GABA(A)R. Alternatively, blocking NMDARs may result in some upstream hindrance to learning the novel rewarding stimulus with an end result of decreased plasticity in the VTA due to ethanol. Some have reported a link between BDNF signaling and NMDA receptor activation with alcohol and amphetamine exposure (Kolb et al., 2005; Fuller et al., 2015). We remain interested in BDNF as an intermediate signaling molecule in the establishment of alcohol dependence, as we have displayed its involvement with opiate dependence (Vargas-Perez et al., 2009). Future experiments could look at NMDA activation or BDNF signaling during the chronic exposure to ethanol.

An increase in GABA release onto VTA DA neurons in brain slices 24 h after a single *in vivo* exposure to ethanol (2 g/kg) has been reported in C57BL/6 and DBA/2 mice (Melis et al., 2002; Wanat et al., 2009), suggesting some plasticity in VTA GABA neuron inhibition of VTA DA neurons that is mediated by NMDARs. Regardless, this state appears to be temporary, as we show here that the sensitivity of VTA GABA neurons to muscimol is restored 7 days after a single exposure to ethanol. Most importantly, DA neuron sensitivity to muscimol was unaffected by a single exposure to ethanol. While there are two studies demonstrating down-regulation of the α1 subunit of the GABA(A)R in the VTA with 12 weeks of chronic ethanol (Ortiz et al., 1995; Charlton et al., 1997), we know of only one study identifiying the specific GABA(A)R subunits expressed in VTA GABA neurons (Tan et al., 2010). A reduction in the levels of the GABA(A)R α1 subunit has been documented in the VTA with chronic ethanol (Ortiz et al., 1995; Charlton et al., 1997; Papadeas et al., 2001). The possibility exists that this reduction is the underlying mechanism for the decreased sensitivity of VTA GABA neurons to muscimol and the lack of sensitivity of VTA DA neurons in mice exposed to chronic ethanol.

Two models of chronic exposure to ethanol were used in this study: twice daily injections of ethanol and CIE in vapor chambers. As mentioned above, we have shown in previous studies that VTA GABA neurons recorded in freely-behaving rats evince hyperexcitability and tolerance to acute ethanol following 2 weeks of twice-daily injections of ethanol (Gallegos et al., 1999). The hyperexcitability of VTA GABA neurons was optimal 24 h after withdrawal from the last injection. Here we show that VTA GABA neurons recorded during withdrawal from twice-daily injections of ethanol resulted in lowered sensitivity to muscimol compared to saline-injected control mice. In effect, lowered sensitivity of GABA(A)Rs on VTA GABA neurons might explain why VTA GABA neurons become hyperexcitable during withdrawal, as they could be experiencing less inhibition from other local circuit GABA neurons or projection GABA neurons from the NAc, ventral pallidum, etc. While chronic injection studies are supportive, it is difficult to determine dependence in rats and mice. The CIE vapor chamber approach enables determinations of dependence, mainly increased alcohol drinking, and eliminates the risk of infection from repeated injections. We show the validity of our alcohol vapor chamber system for establishing alcohol dependence using increased alcohol consumption. Similar results have been shown using DID as a measure of alcohol dependence in other labs (Lopez and Becker, 2005; Dhaher et al., 2008). We compared drinking between ethanol-exposed and naïve air-exposed mice in the DID procedure after withdrawal from 3 weeks of 16 h ON, 8 h OFF CIE, and found that ethanol consumption increased significantly at 24 h and 9 days after withdrawal. After chronic exposure to ethanol vapors, VTA GABA neuron firing rate is also resistant to the inhibitory effects of muscimol, but importantly, this resistance persists after 7 days of withdrawal from chronic ethanol but not acute ethanol exposure. None of these effects were seen in putative VTA DA neurons.

The fact that alterations in muscimol sensitivity are seen only in VTA GABA neurons and not VTA DA neurons strengthens the claim that alcohol's effects on DA in the brain are mediated through VTA GABA neurons. Rather than DA elevation due to alcohol being caused by a direct action on DA neurons, ethanol could disinhibit DA neurons through acting on VTA GABA neurons. We have previously shown that VTA GABA neurons are hyperexcitable during withdrawal from chronic alcohol (Gallegos et al., 1999), which might explain the downregulation of DA neural activity and DA release that is characteristic of alcohol dependence. This decrease of DA activity during withdrawal could contribute to the increased hedonic drive to seek alcohol in order to aleviate the negative consequences of withdrawal. However, it is important to note that VTA GABA neuron baseline firing rate measured in the *ex vivo* slice did not differ between ethanol-exposed and control groups. This finding runs counter to our hypothesis. Thus, we cannot rule out changes in circuit responses to explain increased muscimol resistance in ethanol exposed mice. The discrepancy between *in vivo* and *ex vivo* recordings may be due to the loss of GLUergic and GABAergic inputs due to slicing. Indeed, GLU transmission may be a critical player, as our MK-801 experiments would suggest: mainly, that MK-801 reverses ethanol-induced muscimol resistance 24 h after a single intoxicating dose of ethanol. Regardless, as hypothesized, changes in muscimol sensitivity would still suggest that there could be a decrease in number of GABA(A)Rs, the affinity of GABA(A)Rs for muscimol, a change in subunit composition of the GABA(A)R (e.g., α1 subunit), enhanced GABA(A)R desensitization, or a shift in function of the GABA(A)R, as we have reported in a series of papers with opiate dependence (Laviolette et al., 2004; Vargas-Perez et al., 2009, 2014; Ting-A-Kee et al., 2013). Future studies with perforated patch recordings in dissociated neurons from alcohol-dependent mice and/or chloride imaging will address this issue, particularly with regard to the chloride gradient and the reversal potential of GABA(A)Rs. We are currently developing a procedure to perform perforated-patch clamp recordings in mature animals to address this question.

In conclusion, this study examined the inhibitory effects of the GABA(A)R agonist muscimol on VTA neurons following alcohol exposure. VTA GABA neurons display resistance to muscimol's inhibitory effects following both acute and chronic exposure to ethanol, that persists following chronic exposure, which has implications for the state of the GABA(A)Rs on VTA GABA neurons along the continuum of alcohol dependence. The changes described in this study contribute to altered function of VTA neurons during withdrawal from alcohol that may contribute to the motivation for alcohol-seeking behaviors in dependent individuals.

## Author contributions

SCS and AN designed the experiments. AN, SW, SP, SCS, TW, HP, AP, JO, SIS, and JM performed them and analyzed the data. SCS and AN drafted the manuscript and were responsible for the overall direction of the project and for edits to the manuscript.

### Conflict of interest statement

The authors declare that the research was conducted in the absence of any commercial or financial relationships that could be construed as a potential conflict of interest.
